# Modelling duodenum radiotherapy toxicity using cohort dose-volume-histogram data

**DOI:** 10.1016/j.radonc.2017.04.024

**Published:** 2017-06

**Authors:** Daniel L.P. Holyoake, Marianne Aznar, Somnath Mukherjee, Mike Partridge, Maria A. Hawkins

**Affiliations:** a CRUK/MRC Oxford Institute for Radiation Oncology, Department of Oncology, University of Oxford, United Kingdom; b Nuffield Department of Population Health, University of Oxford, United Kingdom; c Oxford University Hospitals NHS Foundation Trust, United Kingdom

**Keywords:** Pancreatic cancer, Duodenum, Toxicity, Normal tissue, NTCP, Meta-analysis

## Abstract

**Background and purpose:**

Gastro-intestinal toxicity is dose-limiting in abdominal radiotherapy and correlated with duodenum dose-volume parameters. We aimed to derive updated NTCP model parameters using published data and prospective radiotherapy quality-assured cohort data.

**Material and methods:**

A systematic search identified publications providing duodenum dose-volume histogram (DVH) statistics for clinical studies of conventionally-fractionated radiotherapy. Values for the Lyman-Kutcher-Burman (LKB) NTCP model were derived through sum-squared-error minimisation and using leave-one-out cross-validation. Data were corrected for fraction size and weighted according to patient numbers, and the model refined using individual patient DVH data for two further cohorts from prospective clinical trials.

**Results:**

Six studies with published DVH data were utilised, and with individual patient data included outcomes for 531 patients in total (median follow-up 16 months). Observed gastro-intestinal toxicity rates ranged from 0% to 14% (median 8%). LKB parameter values for unconstrained fit to published data were: *n* = 0.070, *m* = 0.46, TD_50(1)_ [Gy] = 183.8, while the values for the model incorporating the individual patient data were *n* = 0.193, *m* = 0.51, TD_50(1)_ [Gy] = 299.1.

**Conclusions:**

LKB parameters derived using published data are shown to be consistent to those previously obtained using individual patient data, supporting a small volume-effect and dependence on exposure to high threshold dose.

## Introduction

Upper-gastrointestinal toxicity remains the principal factor limiting the escalation of radiotherapy dose in the treatment of upper abdominal tumours [1], and irradiation of the duodenum may result in severe, even life-threatening toxicity. Ulceration (which can be acute or late) comes with risk of bleeding or fistula formation and fibrosis (typically late) can lead to stenosis with possible gastric outlet obstruction. The duodenum was not mentioned specifically in the 1991 review of dose-volume by Emami et al. [2] and QUANTEC [3] referred to only one publication reporting specific duodenal toxicity outcomes, with no dose-volume histogram (DVH) data available [4]. Following the QUANTEC publication several studies have shown that risk of toxicity is associated with increased volume of duodenum irradiated to between 25 and 55 Gy. These use data from the treatment of pancreatic cancer [5], [6], [7], [8], liver tumours [9] and para-aortic lymph node irradiation for gynaecological malignancies [10] (Table 1).

**Table 1:** Published duodenum dose-volume parameters predictive of toxicity, with derived dose-volume parameters or volume thresholds (as either absolute or proportional volumes of the duodenum) and associated comparison of proportional incidence of specified toxicity between those patients whose radiotherapy plans achieved or did not achieve this threshold value.

Reference	Cancer site	Concurrent Chemotherapy	Toxicity outcome	Duodenum DVH Parameters	Risk comparison
Huang 2012 [5]	LAPC	Gemcitabine ± erlotinib	Grade ≥ 3 GI	V_25Gy_ 45%	8% vs 48%
Nakamura 2012 [6]	LAPC	Gemcitabine	Grade ≥ 2 GI	D_mean_ 46.1 Gy D_2cm3_ 55.5 Gy	19% vs 57% 21% vs 58%
Cattaneo 2013 [7]	LAPC	Capecitabine or 5FU	Grade ≥ 2 GI	V_40Gy_ 16% V_45Gy_ 2.6%	Not specified
Kelly 2013 [8]	LAPC	Gemcitabine ± 5FU/capecitabine ± EGFRi	Grade ≥ 2 ‘duodenal’	V_55Gy_ 1 cm^3^	9% vs 47%
Yoon 2013 [9]	HCC	None	Grade ≥ 2 ‘gastro-duodenal’	V_35Gy_ 5.4%	9% vs 46%
Verma 2014 [10]	Gynae (PA nodes)	Platinum agents (55 %)	Grade ≥ 2 ‘duodenal’	V_55Gy_ 13.94%	7% vs 49%

DVH: dose-volume histogram; LAPC: locally advanced pancreatic cancer; GI: gastro-intestinal; D_mean:_ mean dose to a structure; D_2cm3_: dose to at least 2cm^3^ of a structure; V_xGy_: volume of structure receiving at least x Gy; 5FU: 5-fluoro-uracil; EGFRi: epidermal growth factor receptor inhibitor; HCC: hepatocellular carcinoma; Gynae: gynaecological malignancies; PA: para-aortic.

Prospective estimation of risk to inform treatment planning for subsequent patients requires derivation of parameters for a normal tissue complication probability (NTCP) model, such as that of Lyman-Kutcher-Burman (LKB) [11]. One study (published as an abstract) has used individual patient data to derive parameters for the LKB model for the duodenum, using the endpoints of gastrointestinal (GI) bleeding following irradiation for liver tumours [12]. A major potential limitation of findings derived from a single cohort of patients is their external generalisability. Results derived using data from multiple institutions and treatment protocols may be more indicative of the true underlying properties of the tissue or organ of interest, and hence be more universally applicable [13]. Two papers have previously attempted to derive NTCP model parameters using published duodenum DVH and toxicity data: Prior and colleagues used published DVH data for the small intestine and duodenum [14], while Elhammali et al. derived LKB parameters for the duodenum using an assumed radiation exposure (homogenous irradiation of 5% of the duodenum by the prescription dose) [15]. Both studies used data from both standard and hypofractionated radiotherapy, however the radiobiology and pathology of upper-GI toxicity is poorly understood, and potentially the mechanisms for normal tissue damage and repair in standard fractionation might follow different pathways than those of extreme hypofractionation.

Our group intended to supplement the available published reports through access to individual patient data from two prospective clinical trials of chemoradiotherapy for locally-advanced pancreatic cancer: the SCALOP study (NCT 01032057, *n* = 74) in which patients were randomised to receive either gemcitabine or capecitabine [16], and the ARCII study (EudraCT 2008-006302-42, *n* = 23) in which patients received concomitant CRT with gemcitabine, cisplatin and nelfinavir (a hypoxia modifier) [17].

The aim of this work is (1) to derive an updated NTCP model for duodenal toxicity in conventional fractionated radiotherapy using available published duodenum DVH data, (2) to test model predictions in two prospective trials delivering chemo-radiation in pancreatic cancer, and (3) further revise the model incorporating prospective trial data.

## Materials and methods

### Updating current NTCP model using published standard fractionation DVH data

A comprehensive literature search was conducted using appropriate keywords and headings (including variants of duodenum, radiotherapy, toxicity, pancreas cancer) in the SCOPUS, EMBASE & MEDLINE databases, limited to reports published in English since 2002. Further suitable publications were identified through existing reviews and results were examined systematically.

### Extraction of DVH data for prospective clinical trial cohorts

For the SCALOP and ARCII datasets the computed tomography (CT) scan, contours, dose cubes and individual patients’ outcomes were available. For the SCALOP cohort the GI tract normal structures had not previously been contoured and were segmented post hoc by one radiation oncologist (DH) according to the recent Radiation Therapy Oncology Group (RTOG) atlas [18], reviewed with a radiologist and a radiation oncologist with an interest in GI oncology. For ARCII the GI tract had previously been contoured but all contours were checked and revised if necessary to achieve consistency with the RTOG guidance. Median DVH statistics for the SCALOP and ARCII clinical trials were derived from the individual patient DVH data. The SCALOP trial had undertaken prospective radiotherapy quality assurance (RTQA) review during the trial including approval of pre-trial benchmark test cases of contouring and planning required before centres were permitted to treat patients in the study [19].

### Equivalent dose calculation and LKB model fitting

To facilitate comparisons between studies the reported duodenum dose-volume parameters were converted to the equivalent dose in 25 fractions (EQD_25#_) (chosen as it was both median and mode among the source cohorts) using an alpha–beta ratio of 4 [20], [21]. For the majority of studies all treatment was delivered in a fixed and consistent number of fractions, but there were some cohorts with mixed numbers of fractions delivered. Verma et al. report that in 7% of their patients a sequential rather than integrated boost was used, but more detail is not provided [10]. In the study by Poorvu et al. sequential dose escalation was titrated to tolerance by normal-tissue constraints, hence in conversions to EQD_25#_ the reference number of fractions for each partial dose-volume in the study was different for each dose level [22]. All source data values are included in Supplementary material for reference.

Cubic splines were fitted to the published DVH data to recreate continuous distributions, which were sampled at 5 Gy intervals to reduce each DVH to a single effective volume *V*_eff_ using the following expression [23]:

(1) $$V_{\text{eff}}=\underset{j}{\sum }\left[{\left(\frac{D_{j}}{D_{\text{max}}}\right)}^{\frac{1}{n}}\Delta V_{j}\right]$$

where $D_{j}$ and $V_{j}$ are the dose and volume of the *j*^th^ element on the DVH, $D_{\text{max}}$ is the maximum dose and *n* is the LKB tissue architecture parameter.

The standard form of the LKB model was adopted [11]:

(2) $$\text{NTCP}=\frac{1}{\sqrt{2\pi }}\int_{-\infty }^{u}e^{-\frac{t^{2}}{2}}\mathrm{dt}$$

where *u* is a function of the “steepness” parameter *m* [11]:

(3) $$u=\frac{D_{\text{max}}-{\text{TD}}_{\text{50}}(V)}{m{\text{TD}}_{\text{50}}(V)}$$

The 50% tolerance dose to a sub-volume of the organ ${\text{TD}}_{\text{50}}(V)$ is found by applying a power law volume effect [11]:

(4) $${\text{TD}}_{\text{50}}(V)=\frac{{\text{TD}}_{\text{50}}(1)}{V_{\text{eff}}^{n}}$$

Values of *n*, *m*, and ${\text{TD}}_{\text{50}}(1)$ (the 50% tolerance dose for irradiation of the whole organ) were found by simultaneously minimising the least square error between the LKB model prediction and all observed clinical outcomes using Levenberg–Marquardt optimisation. Confidence intervals on the fit values obtained were estimated using the leave-one-out method.

The model was firstly fitted using only the published DVH data sources, initially with an unconstrained fit of all three parameters (DuoLKB1). As the resulting value for *n* fell slightly below the confidence intervals for the value derived by Pan et al. (0.09–0.30), the model was fitted again with *n* constrained to ≥0.09 (DuoLKB2). For models DuoLKB1 & DuoLKB2 each data source was treated with equal significance, while for model DuoLKB3 the contribution of each source was weighted according to the number of patients included in that treatment cohort, with an unconstrained fit. The model values were then used to predict the rate of toxicity expected in the clinical trial datasets for which individual patient data were available, and finally the model was fitted again with these data sources included (DuoLKB4).

## Results

The literature search identified 170 results, among which were six publications that reported duodenum dose-volume data and relevant toxicity outcomes and could therefore be included in our analysis [7], [8], [10], [22], [24], [25] (see Table 2). Two of the studies reported data separately for separate cohorts treated using variants of the same treatment protocol and these patient cohorts were considered separately for model fitting. In the analysis by Cattaneo et al., 38 of the 61 patients were treated to a dose of 44.25 Gy in 15 fractions, while the other 23 patients also received a simultaneous integrated boost to a total dose in the range of 48–58 Gy [7]. In the report by Xu et al. 35 of the 76 patients were treated to a dose of 45 Gy in 25 fractions while the other 41 received a boost to 55 Gy in 25 fractions [25]. The SCALOP trial randomised 74 patients to receive capecitabine or gemcitabine as a sensitizer with a radiotherapy dose of 50.4 Gy in 28 fractions delivered conformally [16], whilst ARCII study patients received 50.4 Gy in 28 fractions followed by a sequential boost to 59.4 Gy with cisplatin-gemcitabine-nelfinavir chemotherapy [17].

**Table 2:** Details of publications and clinical trial cohorts with duodenum DVH data available, including those used in this analysis.

Reference	Clinical Data	Patients	mFU, [m]	Cancer site	Radiotherapy Dose-schedule (EQD_25#_ where applicable)	Chemotherapy	Radiotherapy Technique	Toxicity Scale	Grade ≥ 3 Toxicity	Duodenum dose-volume data available
Cattaneo 2013 [7]	NS	61	19	LAPC	45 Gy ± 15 Gy boost in 15 # (EQD_25#_ = 51.9 Gy ± 17.1 Gy)	Capecitabine or 5FU	IMRT	CTCAE v3	12%	D_mean_, V_20Gy,_ V_30Gy,_ V_40Gy,_ V_45Gy_
Kelly 2013 [8]	Retro	106	12	LAPC	50.4 Gy in 28 # (EQD_25#_ = 49.0 Gy)	Gemcitabine ± 5FU/cape ± EGFRi	3D-CRT (75) /IMRT (31)	CTCAE v4	8%	D_mean_, V_40Gy,_ V_45Gy,_ V_50Gy,_ V_55Gy_ V_60Gy_
Xia 2013 [24]	Prosp	33	6	Pancreas	PTV: 50 Gy, GTV: 70 Gy in 20 # (EQD_25#_ = PTV: 53.1 Gy, GTV: 75.0 Gy)	None	Tomo	NS	0%	D_1cc_, D_5cc,_ D_10cc_
Poorvu 2013 [22]	Retro	53	17	Gynae	54 Gy in 30 # (EQD_25#_ = 51.6 Gy)	Cisplatin	IMRT	CTCAE v4	7%	V_55Gy_ & V_60Gy_
Xu 2014 [25]	Retro	76	19	Gynae (PA nodes)	45 Gy ± 10 Gy boost in 25 #	Platinum agents (86%)	IMRT	CTCAE v4	4%	D_mean_, D_Max_, V_35Gy,_ V_40Gy,_ V_50Gy,_V_55Gy,_ D_2cc_, D_5cc_
Verma 2014 [10]	Retro	105	32	Gynae	64 Gy in 25 #	Platinum agents (55%)	IMRT	RTOG	8%	D_Max_, D_2cc_, D_5cc_
Mukherjee 2013 [16]	Prosp	74	12	LAPC	50.4 Gy in 28 # (EQD_25#_ = 49.0 Gy)	Gemcitabine (51%) or capecitabine (49%)	3D-CRT	CTCAE v3	9%	Access to full individual patient DVH data
Wilson 2016 [17]	Prosp	23	14	LAPC	59.4 Gy in 33 # (EQD_25#_ = 55.4 Gy)	Gemcitabine, cisplatin & nelfinavir	3D-CRT/ IMRT	CTCAE v3	14%	Access to full individual patient DVH data
Kim 2009 [33]	Retro	73	11	HCC	36 Gy in 12 #	None	3D-CRT	CTC v2	12%	‘StoDuo’ D_mean_, D_Max_, V_25Gy,_ V_40Gy_
Pan 2003 [12]	Retro	92	7.6	Hepatic	1.5 Gy per # BD with chemo or 1.8 – 3 Gy per # QDS without	Hepatic arterial chemotherapy	3D-CRT	N/A	16%	D_mean_

mFU [m] = median follow-up, months; # = radiotherapy treatment fractions; NS = not specified; Retro = retrospective; Prosp = prospective; LAPC = locally advanced pancreatic cancer; 5FU = 5-fluoro-uracil; cape = capecitabine; IMRT = intensity modulated radiotherapy; 3D-CRT = 3D conformal radiotherapy; Tomo = TomoTherapy; CTC = Common Toxicity Criteria; CTCAE = Common Terminology Criteria for Adverse Events; PA: para-aortic; RTOG = Radiation Therapy Oncology Group Acute Radiation Morbidity Scoring Criteria, StoDuo = combined stomach & duodenum; EQD_25#_ = Equivalent Dose in 25 #, using α/β ratio = 4; BD = twice daily; QDS = four times per day.

The analysis included treatment data for a total of 531 patients with a median of 68 patients (range 23–106) per cohort. Median follow-up duration ranged from 6 to 32 months (median 16 months) and the observed rate of grade ≥ 3 gastrointestinal toxicity ranged from 0 to 14% (median 8%).

**Fig. 1:**
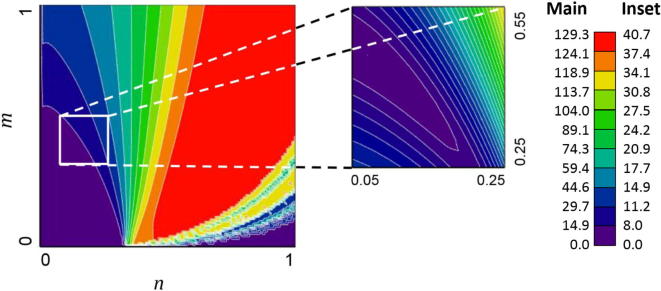
Plot of solution space for model DuoLKB2 (TD_50_(1) value of 142 Gy), showing the low cost (favourable) solutions in purple and the high cost (unfavourable) solutions in red. The degeneracy in *n* and *m* is clearly visible, although the presence of a well-defined minimum valley in solution space is clear. A subset of solution space is shown (inset). The colour bar shows the value of the cost function (note a different scale for the inset plot for clarity).

**Fig. 2:**
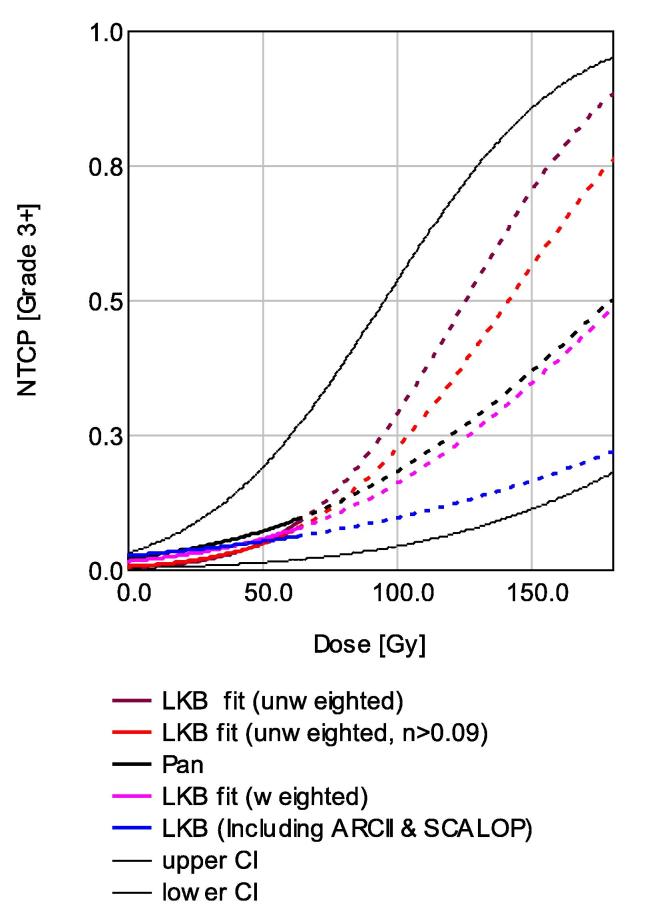
NTCP model of grade ≥ 3 duodenal toxicity fitted to published data for: unconstrained fit (DuoLKB1), fit with constraint *n* ≥ 0.09 (DuoLKB2), fit weighted according to cohort size (DuoLKB3), weighted fit including ARCII and SCALOP data (DuoLKB4), and the model parameters as published by Pan et al. Confidence intervals for the fitting process show the envelope of solutions given using a leave-one-out error estimation. Curves are shown by dotted lines where the model extrapolates beyond the region supported by the data.

The LKB model parameter values derived using the different model fits are detailed in Table 3. The solution space for DuoLKB2 (LKB function with a TD_50_ (1) value of 142 Gy) is visualised in Fig. 1. Fig. 2 shows a plot of the four models (DuoLKB1-4) and the model derived by Pan et al. Confidence limits are shown for the unconstrained model, derived using leave-one out cross-validation.

**Table 3:** Results of LKB model fitting. Mean values for parameters are shown with 95% confidence intervals estimated by refitting all data using the leave-one-out method (in each refitting of DuoLKB2 the optimal value for parameter *n* was precisely 0.09).

Model	Data source	Fitting details	*n*	*m*	TD_50_ (1) [Gy]
DuoLKB1	Published data	Unconstrained, unweighted	0.068 (0.060–0.076)	0.36 (0.30–0.43)	125.9 (63.1–188.7)
DuoLKB2	Published data	Constraint: *n* > 0.09	0.090 (0.090–0.090)	0.39 (0.32–0.46)	141.8 (36.7–246.9)
DuoLKB3	Published data	Unconstrained, weighted	0.070 (0.061–0.079)	0.46 (0.40–0.52)	183.8 (122.1–245.5)
DuoLKB4	Published & IPD	Unconstrained, weighted	0.193 (0.147–0.239)	0.51 (0.47–0.55)	299.1 (242.1–356.1)

IPD: Individual Patient Data.

The proportional incidence of grade 3 gastro-intestinal toxicity observed in the SCALOP trial was 8.8%, and the values predicted by the three models DuoLKB1, 2 and 3 were 7.4%, 7.6% and 7.0% respectively. For the ARCII trial, the observed toxicity incidence was 14.3%, while the incidence predicted by the model fits were 8.9%, 9.1% and 7.8% respectively.

**Fig. 3:**
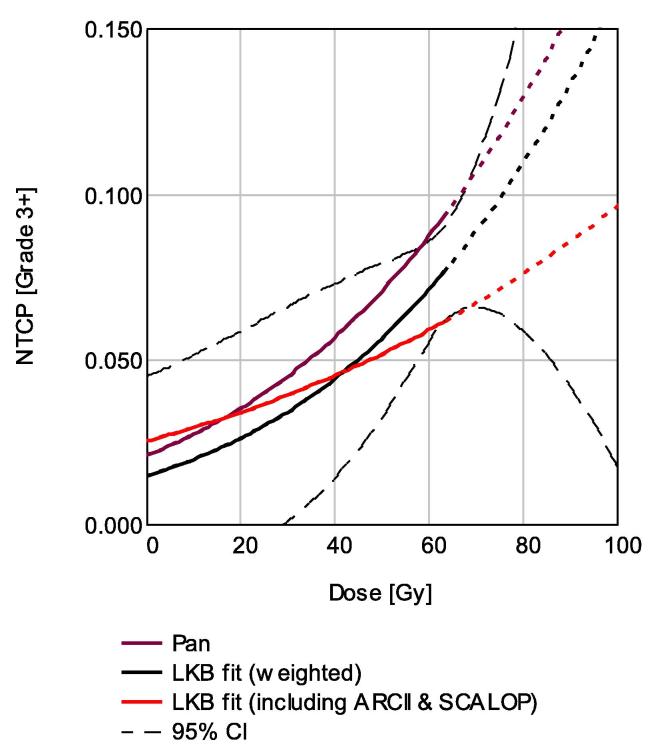
NTCP model of duodenal toxicity published by Pan, and fitted to published data either excluding (DuoLKB3) or including (DuoLKB4) the ARCII and SCALOP trials, weighted according to cohort size. 95% confidence intervals are shown for the fit excluding the ARCII and SCALOP trials.

Fig. 3 shows the model derived when incorporating the clinical trial datasets (DuoLKB4), along with 95% confidence estimates for this curve, demonstrating the uncertainty that exists outside of the observed data range.

## Discussion

We have identified publications with clinical duodenum DVH data which we have used to fit the LKB model and derive parameter values that have consistency with those derived by existing publications using individual patient data. The derived values were especially similar to previous results when the sources were weighted according to the number of patients in each cohort, as highlighted in Fig. 2. We have then used these parameters to predict toxicity within two clinical trials of pancreatic cancer chemo-radiotherapy, with accurate results for one study but not the other. We believe this to be the first time this iteration for developing an NTCP model has been used, and think it is likely that the particular chemotherapy combination used in the ARCII trial (concurrent gemcitabine, cisplatin and nelfinavir) explains the observed toxicity in this study being higher than is predicted by the model. When we incorporate the data from these clinical trials the model fit is less consistent with existing literature, however this comprises the largest meta-analysis of this type to have been conducted. This model (DuoLKB4) is based on the pooled data regarding treatment of over five hundred patients and is therefore the model we would suggest be adopted for clinical practice.

The values derived for the parameter *n* are consistent with a small volume effect for the toxicity endpoint. Low values of *n* increase the dependence of the outcome on the maximum dose received by the tissue and are seen for tissues (or endpoints) where the functional subunits (FSU’s) are arranged in a serial manner. An example is myelitis in the spinal cord, where the volume of the organ affected (along the axial length of the cord) is of little consequence [26]. Our results suggest a similar behaviour for the duodenum, where the maximum exposure dose is more important than the affected volume of the organ at risk.

Pan and colleagues had derived LKB model values for the duodenum using retrospective analysis of 92 patients treated with conformal radiotherapy [12]. The patients received either 1.5 Gy twice-daily with intrahepatic arterial chemotherapy or 1.8–3.0 Gy four times per day without chemotherapy, hence for analysis the authors converted these to effective doses at 2 Gy per fraction (EQD_2Gy_). The LKB values derived were *n* = 0.12 (0.09–0.30), *m* = 0.49 (0.36–0.61) and TD_50_(1) = 180 Gy (69% CI ≈ 100–>200 Gy), suggesting a small volume effect and shallow dose-NTCP curve. When the value of *n* was constrained to ≥0.09, the other fitted values also shifted closer to those of Pan et al., but with no change in the goodness of fit, suggesting these values are equally appropriate to our data. Interestingly, the values for DuoLKB3 were even closer to those of Pan et al.

When analysing conventionally fractionated chemoradiotherapy in pancreatic cancer Murphy et al. did not identify any significant associations of toxicity with specific duodenum dose-volume parameters, though saw a trend for association with generalised equivalent uniform dose (gEUD, a DVH-reduction parameter closely related to V_eff_ in the LKB model [27], [28], [29]) [30]. They subsequently derived LKB parameters for the duodenum using a cohort of 73 patients treated with single-fraction SBRT for inoperable pancreatic cancer (*n* = 0.12, *m* = 0.23, and TD_50_ (1)= 24.6 Gy) [31]. The authors acknowledged that comparing single-fraction treatment with conventional fractionation regimens is challenging, however the EQD_2Gy_ for 24.6 Gy in a single fraction is 117.3 Gy (alpha–beta 4 Gy), a value not dissimilar to those established by other authors and ourselves.

In their meta-analysis Prior et al. incorporated published duodenum DVH and toxicity data from four studies (two using conventional fractionation, also used in this investigation, and two using hypofractionated radiotherapy) encompassing 312 patients [14]. A model was derived partly using small-bowel homogenous irradiation tolerance data from Burman et al. [32], hence they were unable to derive a value for *n*, and this may explain the difference between the LKB values they have fitted (*m* = 0.21 ± 0.05 and TD_50_ (1)= 60.9 ± 7.9 Gy) and ours.

Elhammali et al. collated toxicity data from 16 human studies (and two canine studies) involving a total of 1160 patients and used regression analysis to show that dose was the only significant predictor of toxicity among the studies they analysed [15]. The authors went on to derive LKB parameters *n* = 0.38–0.63, *m* = 0.48–0.49, and TD_50_ (1) = 35–95 Gy, however as the majority of publications they examined did not report treatment DVH data the authors had resorted to an assumption that across all studies 1–5% of the duodenum was exposed to the prescription dose. Their values for *n* are higher and their values for TD_50_ (1) are considerably lower than those found in other studies (including ours). The assumption of volume made by the authors is not likely to reflect the true exposure of the duodenum in these patients, particularly when some cohorts included single-fraction intraoperative radiotherapy and others were preclinical animal studies, and these further particularities limit the applicability of these results to conventional clinical external beam irradiation.

A key limitation of our own study is the persistently small number of somewhat heterogenous studies that provide suitable data, though our results are potentially strengthened by the addition of data derived directly from the complete DVH for the individual patients treated in the SCALOP and ARCII trials, and the coherent use of similarly fractionated studies. One publication that was found and reviewed provided DVH data only for a combined stomach-duodenum structure [33] hence these values were not incorporated into the model fitting, while Pan et al. reported only mean dose for the duodenum [12]. While there is some degeneracy of the parameter values derived in our model fitting there is also a well-defined minimum ‘valley’ as shown in Fig. 1. The confidence intervals for our parameter values are broad and the proportion of each of our models that is extrapolated beyond the observed data should be interpreted with caution. We acknowledge that the spline-fitting method we have used to recreate the DVH’s from reported data points may lack precision when few data are provided. We also appreciate further uncertainty exists in in the conversion of the varying dose-fractionation schedules, and in the influence of the differing chemotherapy drugs and combination regimens on the behaviour of duodenal radiotherapy toxicity.

For an organ to be studied rigorously, it must be defined consistently. Detailed guidance on the delineation of the duodenum has now been published in the recent RTOG upper GI atlas, but the authors of this guideline noted that the fourth part of the duodenum was frequently missed by the contributing clinicians, meaning that existing data relating to this organ may be affected by this inconsistency in anatomical delineation [18]. While the duodenum is less mobile than other parts of the small bowel, large inter-fractional variations in volume still occur and which can lead to significant differences in delivered dosimetry compared to that which is planned [34], [35], [36]. Very few publications have investigated the delivered or accumulated dose to upper GI organs [37], and the data used here rely on planned dose as a surrogate.

The perceived benefits of the LKB model include the rational interpretation that can be made of the parameter values, relating to tissue architecture, dose–response gradient and tolerance dose. However, the LKB model originates from a time of more homogenous dose distribution across target structures and normal-tissues, and the DVH-reduction step may be inappropriate for the modern era of highly modulated radiotherapy dose depositions as the detail of the shape of the DVH will be obscured [38]. Furthermore, in hollow organs such as the gastrointestinal tract the tissue of interest is only a thin layer surrounding a variable amount of contents and dose-surface-maps may therefore offer greater insight into the causality of toxicity in hollow or tubular organs [37], [39].

Gastrointestinal toxicity outcomes and their relationships to the relevant tissues are complex, and there is subtle variation in the endpoints defined by the source publications utilised here. Many of the relevant symptoms that indicate radiation toxicity (nausea, vomiting, anorexia, abdominal pain) could arise from damage to the other tissues of the abdomen (particularly the stomach and small bowel) even if the duodenum were entirely spared, or could result from systemic therapy or the underlying disease. Some analyses have confined their study to outcomes with physical evidence of toxicity, such as ulceration or bleeding in the organ of interest, proven using endoscopy. To us this seems an oversimplification, which may overlook other features of duodenal toxicity that also cause patient morbidity and may impair outcomes if they were to impede the delivery of a prescribed course of radiotherapy.

While results of dosimetry-toxicity analysis have differed between studies the predictive value of the duodenum V_55Gy_ has been reproduced by independent investigators [8], [10]. Similarly while there is variability in the values that have been derived for the LKB model by the various publications considered here, there is some consistency in the ranges of results observed, and the results of our meta-analysis are closer to those found in studies of individual patient data [12] than in the two other attempted meta-analyses [14], [15]. This we attribute to the use of collated rather than assumed volume data, and the exclusion of possibly confounding hypofractionated radiotherapy data.

## Conclusions

We have successfully derived parameters for the LKB model for the duodenum using reconstructed DVH data from a set of publications reporting clinical toxicity outcomes after irradiation of upper abdominal tumours, which show some consistency with values derived using individual patient data. These parameters can be used to understand the dependence of toxicity in this organ on dose and volume and potentially predict toxicity risk in a patient cohort, but work in this field is restricted by a limited availability of source data and the complexity of the outcome of interest.

## Conflict of interest statement

The authors declare that they have no competing or conflicting interests.

## References

[1] Ben-Josef E., Schipper M., Francis I.R. (2012). A phase I/II trial of intensity modulated radiation (IMRT) dose escalation with concurrent fixed-dose rate gemcitabine (FDR-G) in patients with unresectable pancreatic cancer. Int J Radiat Oncol Biol Phys.

[2] Emami B., Lyman J., Brown A. (1991). Tolerance of normal tissue to therapeutic irradiation. Int J Radiat Oncol Biol Phys.

[3] Kavanagh B.D., Pan C.C., Dawson L.A. (2010). Radiation dose-volume effects in the stomach and small bowel. Int J Radiat Oncol Biol Phys.

[4] Hamilton C.R., Horwich A., Bliss J.M., Peckham M.J. (1987). Gastrointestinal morbidity of adjuvant radiotherapy in stage I malignant teratoma of the testis. Radiother Oncol.

[5] Huang J., Robertson J.M., Ye H., Margolis J., Nadeau L., Yan D. (2012). Dose-volume analysis of predictors for gastrointestinal toxicity after concurrent full-dose gemcitabine and radiotherapy for locally advanced pancreatic adenocarcinoma. Int J Radiat Oncol Biol Phys.

[6] Nakamura A., Shibuya K., Matsuo Y. (2012). Analysis of dosimetric parameters associated with acute gastrointestinal toxicity and upper gastrointestinal bleeding in locally advanced pancreatic cancer patients treated with gemcitabine-based concurrent chemoradiotherapy. Int J Radiat Oncol Biol Phys.

[7] Cattaneo G.M., Passoni P., Longobardi B. (2013). Dosimetric and clinical predictors of toxicity following combined chemotherapy and moderately hypofractionated rotational radiotherapy of locally advanced pancreatic adenocarcinoma. Radiother Oncol.

[8] Kelly P., Das P., Pinnix C.C. (2013). Duodenal toxicity after fractionated chemoradiation for unresectable pancreatic cancer. Int J Radiat Oncol Biol Phys.

[9] Yoon H., Oh D., Park H.C. (2013). Predictive factors for gastroduodenal toxicity based on endoscopy following radiotherapy in patients with hepatocellular carcinoma. Strahlenther Onkol.

[10] Verma J., Sulman E.P., Jhingran A. (2014). Dosimetric predictors of duodenal toxicity after intensity modulated radiation therapy for treatment of the para-aortic nodes in gynecologic cancer. Int J Radiat Oncol Biol Phys.

[11] Kutcher G.J., Burman C. (1989). Calculation of complication probability factors for non-uniform normal tissue irradiation: the effective volume method. Int J Radiat Oncol Biol Phys.

[12] Pan C.C., Dawson L.A., McGinn C.J., Lawrence T.S., Ten Haken R.K. (2003). Analysis of radiation-induced gastric and duodenal bleeds using the Lyman-Kutcher-Burman model. Int J Radiat Oncol Biol Phys.

[13] Semenenko V.A., Li X.A. (2008). Lyman-Kutcher-Burman NTCP model parameters for radiation pneumonitis and xerostomia based on combined analysis of published clinical data. Phys Med Biol.

[14] Prior P., Tai A., Erickson B., Li X.A. (2014). Consolidating duodenal and small bowel toxicity data via isoeffective dose calculations based on compiled clinical data. Pract Radiat Oncol.

[15] Elhammali A., Patel M., Weinberg B. (2015). Late gastrointestinal tissue effects after hypofractionated radiation therapy of the pancreas. Radiat Oncol.

[16] Mukherjee S., Hurt C.N., Bridgewater J. (2013). Gemcitabine-based or capecitabine-based chemoradiotherapy for locally advanced pancreatic cancer (SCALOP): a multicentre, randomised, phase 2 trial. Lancet Oncol.

[17] Wilson J.M., Fokas E., Dutton S.J. (2016). ARCII: a phase II trial of the HIV protease inhibitor Nelfinavir in combination with chemoradiation for locally advanced inoperable pancreatic cancer. Radiother Oncol.

[18] Jabbour S.K., Hashem S.A., Bosch W. (2014). Upper abdominal normal organ contouring guidelines and atlas: a Radiation Therapy Oncology Group consensus. Pract Radiat Oncol.

[19] Fokas E., Clifford C., Spezi E. (2015). Comparison of investigator-delineated gross tumor volumes and quality assurance in pancreatic cancer: Analysis of the pretrial benchmark case for the SCALOP trial. Radiother Oncol.

[20] Dische S., Saunders M.I., Sealy R. (1999). Carcinoma of the cervix and the use of hyperbaric oxygen with radiotherapy: a report of a randomised controlled trial. Radiother Oncol.

[21] Deore S.M., Shrivastava S.K., Supe S.J., Viswananthan P.S., Dinshaw K.A. (1993). α/β value and importance of dose per fraction for the late rectal and recto-sigmoid complications. Strahlenther Onkol.

[22] Poorvu P.D., Sadow C.A., Townamchai K., Damato A.L., Viswanathan A.N. (2013). Duodenal and other gastrointestinal toxicity in cervical and endometrial cancer treated with extended-field intensity modulated radiation therapy to paraaortic lymph nodes. Int J Radiat Oncol Biol Phys.

[23] Kutcher G.J., Burman C., Brewster L., Goitein M., Mohan R. (1991). Histogram reduction method for calculating complication probabilities for three-dimensional treatment planning evaluations. Int J Radiat Oncol Biol Phys.

[24] Xia T., Chang D., Wang Y., Li J., Wu W., Zhu F. (2013). Dose escalation to target volumes of helical tomotherapy for pancreatic cancer in the phase 1–2 clinical trial. Int J Radiat Oncol Biol Phys.

[25] Xu K.M., Rajagopalan M.S., Kim H., Beriwal S. (2015). Extended field intensity modulated radiation therapy for gynecologic cancers: Is the risk of duodenal toxicity high?. Pract Radiat Oncol.

[26] Marks L.B., Yorke E.D., Jackson A. (2010). Use of normal tissue complication probability models in the clinic. Int J Radiat Oncol Biol Phys.

[27] Niemierko A. (1997). Reporting and analyzing dose distributions: a concept of equivalent uniform dose. Med Phys.

[28] Niemierko A. A generalized concept of equivalent uniform dose (EUD), Med Phys. 1999;26: 1101. 10.1118/1.598063 9029544

[29] Wang J.Z., Mayr N.A., Yuh W.T. (2008). Behind EUD. Acta Oncol.

[30] Murphy J.D., Adusumilli S., Griffith K.A. (2007). Full-dose gemcitabine and concurrent radiotherapy for unresectable pancreatic cancer. Int J Radiat Oncol Biol Phys.

[31] Murphy J.D., Christman-Skieller C., Kim J., Dieterich S., Chang D.T., Koong A.C. (2010). A dosimetric model of duodenal toxicity after stereotactic body radiotherapy for pancreatic cancer. Int J Radiat Oncol Biol Phys.

[32] Burman C., Kutcher G.J., Emami B., Goitein M. (1991). Fitting of normal tissue tolerance data to an analytic function. Int J Radiat Oncol Biol Phys.

[33] Kim H., Lim D.H., Paik S.W. (2009). Predictive factors of gastroduodenal toxicity in cirrhotic patients after three-dimensional conformal radiotherapy for hepatocellular carcinoma. Radiother Oncol.

[34] Singh A.K., Tierney R.M., Low D.A. (2006). A prospective study of differences in duodenum compared to remaining small bowel motion between radiation treatments: implications for radiation dose escalation in carcinoma of the pancreas. Radiat Oncol.

[35] Witztum A, Warren S, Holyoake D, Partridge M, Mukherjee S, Hawkins MA. EP-1474: The dosimetric effect of interfraction motion on the duodenum in pancreatic radiotherapy Radiother Oncol. 2015;115 S800-S1.

[36] Houweling A.C., Fukata K., Kubota Y. (2016). The impact of interfractional anatomical changes on the accumulated dose in carbon ion therapy of pancreatic cancer patients. Radiother Oncol.

[37] Scaife J.E., Thomas S.J., Harrison K. (2015). Accumulated dose to the rectum, measured using dose-volume histograms and dose-surface maps, is different from planned dose in all patients treated with radiotherapy for prostate cancer. Br J Radiol..

[38] Gulliford S.L., Partridge M., Sydes M.R., Webb S., Evans P.M., Dearnaley D.P. (2012). Parameters for the Lyman Kutcher Burman (LKB) model of normal tissue complication probability (NTCP) for specific rectal complications observed in clinical practise. Radiother Oncol..

[39] Witztum A., George B., Warren S., Partridge M., Hawkins M.A. (2016). Unwrapping 3D complex hollow organs for spatial dose surface analysis. Med Phys.

