# Supplementary material for: Modelling duodenum radiotherapy toxicity using cohort dose-volume-histogram data
Source: Radiother Oncol. 2017 Jun;123(3):431–7. doi: 10.1016/j.radonc.2017.04.024 (PMC5486774; doi:10.1016/j.radonc.2017.04.024)
Supplement: Supplementary data [file mmc1.docx]

Supplementary data table: Published DVH data values for studies included in this analysis

| Reference | Cattaneo  2013 (7) | | |  | Kelly  2013 (8) | | |  | Xia  2013 (24) |  | Poorvu 2013 (22) |  | Xu  2014 (25) | | |  | Verma 2014 (10) |
| --- | --- | --- | --- | --- | --- | --- | --- | --- | --- | --- | --- | --- | --- | --- | --- | --- | --- |
| Data | Mean (SD) | | |  | Mean (SD) | | |  | Mean (SD) |  | Mean (range) |  | Mean / median (range) | | |  |  |
| Cohort | Group 1 (SIB) | Group 2 | Weighted average |  | G0-1 toxicity | G2-5 toxicity | Weighted average |  |  |  |  |  | High dose | Low dose | All patients |  |  |
| Patients | 23 | 38 | 61 |  | 90 | 16 | 106 |  | 25 |  | 53 |  | 41 | 35 | 76 |  | 105 |
| Volume [cm^3^] | 69  (36.4) | 76  (58) | 73.4 |  |  |  |  |  |  |  |  |  | 73.9  (21.2-148.4) | 94.3  (26.2-174.9) | 83.2  (21.2-174.9) |  |  |
| D_Mean_  [Gy] | 21  (6.5) | 22  (7.2) | 21.6 |  | 36  (14) | 44  (9) | 41 |  |  |  |  |  |  |  |  |  |  |
| V_20Gy_  [cm^3^] | 33  (23.7) | 34.5  (18.8) | 33.9 |  |  |  |  |  |  |  |  |  |  |  |  |  |  |
| V_30Gy_  [cm^3^] | 19.5  (15.4) | 24  (15.9) | 22.3 |  |  |  |  |  |  |  |  |  |  |  |  |  |  |
| V_35Gy_ [cm^3^] |  |  |  |  |  |  |  |  |  |  |  |  | 47.5  (0.3-100.9) | 36.2  (0-90.7) | 41.2  (0-100.9) |  |  |
| V_40Gy_  [cm^3^] | 11  (9.8) | 13  (11.9) | 12.2 |  | 46  (34) | 44  (24) | 46 |  |  |  |  |  | 41.4  (0-90.0) | 28.3  (0-77.3) | 34.5  (0-90.0) |  |  |
| V_45Gy_  [cm^3^] | 3.5  (3.2) | 0.4  (1) | 1.6 |  | 42  (33) | 41  (23) | 42 |  |  |  |  |  |  |  |  |  |  |
| V_50Gy_  [cm^3^] |  |  |  |  | 34  (30) | 34  (23) | 34 |  |  |  |  |  | 4.0  (0-21.9) | 1.0  (0-20.0) | 2.6  (0-21.9) |  |  |
| V_55Gy_  [cm^3^] |  |  |  |  | 2  (11) | 5  (10) | 2 |  |  |  | 1.5  (0-14.2) |  | 0.8  (0-10.6) | 0 | 0.4  (0-10.6) |  |  |
| V_60Gy_  [cm^3^] |  |  |  |  | 0.3  (1.4) | 1.7  (3.0) | 0.5 |  |  |  | 0.4  (0-7.6) |  |  |  |  |  |  |
| D_10cc_  [Gy] |  |  |  |  |  |  |  |  | 33.39 (1.53) |  |  |  |  |  |  |  |  |
| D_5cc_  [Gy] |  |  |  |  |  |  |  |  | 38.91 (1.41) |  |  |  | 48.8  (31.1-57.3) | 34.0  (0-51.9) | 42.0  (0-57.3) |  |  |
| D_2cc_  [Gy] |  |  |  |  |  |  |  |  |  |  |  |  | 50.1  (31.3-58.3) | 34.9  (0-52.3) | 43.1  (0-58.3) |  | 59.6 |
| D_1cc_  [Gy] |  |  |  |  |  |  |  |  | 47.38  (1.19) |  |  |  |  |  |  |  | 57.6 |
| D_Max_  [Gy] |  |  |  |  |  |  |  |  |  |  |  |  | 53.8  (38.0-63.2) | 37.3  (0-53.3) | 46.2 (0-63.2) |  | 63.4 |

**References**

[7] Cattaneo GM, Passoni P, Longobardi B, et al. Dosimetric and clinical predictors of toxicity following combined chemotherapy and moderately hypofractionated rotational radiotherapy of locally advanced pancreatic adenocarcinoma Radiother Oncol. 2013;108(1):66-71.

[8] Kelly P, Das P, Pinnix CC, et al. Duodenal toxicity after fractionated chemoradiation for unresectable pancreatic cancer Int J Radiat Oncol Biol Phys. 2013;85(3):e143-9.

[10] Verma J, Sulman EP, Jhingran A, et al. Dosimetric predictors of duodenal toxicity after intensity modulated radiation therapy for treatment of the para-aortic nodes in gynecologic cancer Int J Radiat Oncol Biol Phys. 2014;88(2):357-62.

[22] Poorvu PD, Sadow CA, Townamchai K, Damato AL, Viswanathan AN. Duodenal and other gastrointestinal toxicity in cervical and endometrial cancer treated with extended-field intensity modulated radiation therapy to paraaortic lymph nodes Int J Radiat Oncol Biol Phys. 2013;85(5):1262-8.

[24] Xia T, Chang D, Wang Y, Li J, Wu W, Zhu F. Dose Escalation to Target Volumes of Helical Tomotherapy for Pancreatic Cancer in the Phase 1-2 Clinical Trial Int J Radiat Oncol Biol Phys. 2013;87(2):S303-S.

[25] Xu KM, Rajagopalan MS, Kim H, Beriwal S. Extended field intensity modulated radiation therapy for gynecologic cancers: Is the risk of duodenal toxicity high? Pract Radiat Oncol. 2015;5(4):e291-7.
